# 

**Published:** 1892-06

**Authors:** 


### CONTENTS.

ORIGINAL ARTICLES—The Present Status of Obstetrics and Gynæcology —By C. D. Palmer, M. D., Cincinnati, Ohio....315
The Influence of Civilization and Social Life Upon Women, as Viewed from a Medical Standpoint—By C. D. Hurt, M. D., Cedartown, Ga....322
Abortion in an Hour—By C. H. Harris, M. D., Cedartown, Ga....329
CORRESPONDENCE—Our New York Letter....333–336
Observation Abroad....336–343
SOCIETY NOTES—Clinical Society of Maryland....344–350
BOOK REVIEWS....351–352
SELECTIONS AND ABSTRACTS—Treatment of Tape-worm....321
An Interesting Case....343
Eye Troubles in Pregnancy....352
Bromide of Strontium in Epilepsy....353
Borax in Epilepsy....353
A Neglected Method—Reunion of Cut-off Fingers....354
Amenorrhœa in School Girls—Penetrating Wounds....355
International Periodical Congress of Gynæcology and Obstetrics....356
Chloride of Iron in Typhoid Fever....356
Acute Rheumatism....357
A New York Malpractice Case—Treatment of Hemorrhoids....358
Pregnancy after Double Oophorectomy....358
EDITORIAL—A Growing Evil Which Should be Remedied....359
Micro-Organisms and the Sweat....361
PRESCRIPTION DEPARTMENT....365–366
Carbuncles and Boils—Cold in the Head—Chronic Gastritis—Catarrhal Affections—Otorrhœa—Chronic Rheumatism—For Habitual Constipation—For Dry, Ringing Cough of Influenza—Burns—Morning Laxative—Dyspeptic Vertigo.
SPECIAL NOTES....363–364

